# Separating Features From Functionality in Vaccination Apps: Computational Analysis

**DOI:** 10.2196/36818

**Published:** 2022-10-11

**Authors:** George Shaw Jr, Devaki Nadkarni, Eric Phann, Rachel Sielaty, Madeleine Ledenyi, Razaan Abnowf, Qian Xu, Paul Arredondo, Shi Chen

**Affiliations:** 1 Public Health Sciences School of Data Science University of North Carolina Charlotte, NC United States; 2 Public Health Sciences University of North Carolina at Charlotte Charlotte, NC United States; 3 Department of Computer Science University of North Carolina at Charlotte Charlotte, NC United States; 4 Department of Language and Culture Studies University of North Carolina at Charlotte Charlotte, NC United States; 5 Department of Biological Sciences University of North Carolina at Charlotte Charlotte, NC United States; 6 Department of Global Studies Belk College of Business University of North Carolina at Charlotte Charlotte, NC United States; 7 School of Communications Elon University Elon, NC United States; 8 School of Data Science University of North Carolina Charlotte, NC United States

**Keywords:** vaccines, mobile health, mHealth, principal component analysis, PCA, k-means clustering, information exchange, mobile phone

## Abstract

**Background:**

Some latest estimates show that approximately 95% of Americans own a smartphone with numerous functions such as SMS text messaging, the ability to take high-resolution pictures, and mobile software apps. Mobile health apps focusing on vaccination and immunization have proliferated in the digital health information technology market. Mobile health apps have the potential to positively affect vaccination coverage. However, their general functionality, user and disease coverage, and exchange of information have not been comprehensively studied or evaluated computationally.

**Objective:**

The primary aim of this study is to develop a computational method to explore the descriptive, usability, information exchange, and privacy features of vaccination apps, which can inform vaccination app design. Furthermore, we sought to identify potential limitations and drawbacks in the apps’ design, readability, and information exchange abilities.

**Methods:**

A comprehensive codebook was developed to conduct a content analysis on vaccination apps’ descriptive, usability, information exchange, and privacy features. The search and selection process for vaccination-related apps was conducted from March to May 2019. We identified a total of 211 apps across both platforms, with iOS and Android representing 62.1% (131/211) and 37.9% (80/211) of the apps, respectively. Of the 211 apps, 119 (56.4%) were included in the final study analysis, with 42 features evaluated according to the developed codebook. The apps selected were a mix of apps used in the United States and internationally. Principal component analysis was used to reduce the dimensionality of the data. Furthermore, cluster analysis was used with unsupervised machine learning to determine patterns within the data to group the apps based on preselected features.

**Results:**

The results indicated that readability and information exchange were highly correlated features based on principal component analysis. Of the 119 apps, 53 (44.5%) were iOS apps, 55 (46.2%) were for the Android operating system, and 11 (9.2%) could be found on both platforms. Cluster 1 of the k-means analysis contained 22.7% (27/119) of the apps; these were shown to have the highest percentage of features represented among the selected features.

**Conclusions:**

We conclude that our computational method was able to identify important features of vaccination apps correlating with end user experience and categorize those apps through cluster analysis. Collaborating with clinical health providers and public health officials during design and development can improve the overall functionality of the apps.

## Introduction

### Background

IT has revolutionized all aspects of the world, including our health care system. IT has enhanced the overall efficiency and accessibility of patient care [1]. Smartphones are a type of IT that has become important within health care [2]. Some of the latest estimates show that approximately 95% of Americans own a smartphone with numerous functio0ns such as texting, the ability to take high-resolution pictures, and mobile software apps [3]. Owners of smartphones also use the available functions to manage various facets of their health [4]. Today, mobile health (mHealth) technology plays a crucial role in providing quality health care services by improving health outcomes and facilitating health care access. Istepanian et al [5] defined mHealth as mobile computing, medical sensor, and communication technologies designed for health care. The use of mHealth apps provides an efficient way for patients to share their medical information with providers, improves the collection of real-time health information, and supports vaccine uptake [6].

A significant concern that is often communicated by mHealth app users is data privacy. In the United States, the Health Insurance Portability and Accountability Act (HIPAA) ensures that health care entities provide adequate measures to protect patient data. Many consumer-based apps that track and monitor health data are not HIPAA compliant. Health care stakeholders in the United States recommend that mobile apps designed for health care be HIPAA compliant [2,7]. Another impediment faced by mHealth apps in the current dynamic immunization practice is a 1-sided vaccine delivery system or lack of bidirectional information exchange. There exists the opportunity to leverage mHealth apps as tools to support public navigation of complex health systems and promote bidirectional communication of information between the public and health providers. Factors such as lack of health care access, fragmented vaccine provider systems, and low vaccine literacy can lead to undervaccination in the community [8]. Moreover, vaccine hesitancy can further reduce vaccine uptake among populations and undermine previous gains in eradicating communicable diseases [6].

Vaccine hesitancy—the delay or refusal to be vaccinated despite available vaccination services—is a complex phenomenon that involves emotional, cultural, social, spiritual, and political factors [9,10]. When considering vaccine hesitancy and decision-making, parental vaccine hesitancy stems from a variety of reasons, and there is no one-size-fits-all *type* of parent who chooses to forgo vaccinating their child [8,11,12]. Suspected autism side effects, religious reasons, concerns over the “newness” of the vaccine, and inaccurate portrayal of vaccines in various media outlets are common factors that influence parental vaccine hesitancy [6,13]. The recent resurgence of outbreaks of whooping cough and measles in children is a prime example of vaccination refusal associated with the resurgence of preventable communicable diseases in communities [13]. Although vaccination mHealth apps are attempting to address this issue, current results are mixed [14].

### Rationale and Aim

Results from a recent systematic review reported a lack of evidence supporting the use of vaccination apps geared toward children, as shown through vaccination uptake, knowledge, and decision-making [15]. Another systematic review reported that mHealth improved vaccination uptake among adults and children; however, there is inconclusive evidence that digital solutions will achieve optimal vaccination coverage [16]. Barriers such as technology hesitancy, complicated app navigation, and difficult app features can compromise vaccination app use. Security and storage compliance associated with HIPAA, along with transmission and protection of private health data collected through vaccination apps, is another pressing concern.

The primary aim of our study was to develop a computational method to explore the descriptive, usability, information exchange, and privacy features of various vaccination apps, which can inform vaccination app design. We also aimed to assess these apps using a content analysis approach and identify potential flaws in app functionality. This study analyzed these data according to their respective operating platforms and collectively. The content analysis approach used was adapted from previous studies [17,18].

## Methods

### Definition and Identification of Vaccination-Related Apps

For this study, vaccination-related apps were operationalized as apps that allowed tracking, scheduling, and general dissemination of vaccination information [6,19]. Apps were included if they were found on the Google Play Store and the Apple App Store. The query terms “vaccination,” “immunization,” “vaccine,” “immunization schedule,” and “vaccination schedule” were used in the search process to generate our sample of apps. English-language apps and English-language apps with a second language were both included. We did not search any cell phone manufacturer app stores (eg, Samsung Galaxy Store) as Google Play and the Apple App Store are prominent web-based marketplaces used by Android and iOS smartphone users to download apps. Apps characterized as sideload apps and homebrew apps were excluded. Sideload apps are apps that have not been certified to be included within an app store. We characterized homebrew apps as apps that can be downloaded using a computer terminal.

The search and selection process for the apps was conducted from March to May 2019. We identified a total of 211 apps across both platforms, with iOS and Android representing 62.1% (131/211) and 37.9% (80/211) of the apps, respectively. As iOS apps represented more than half of the apps collected, we chose a random sample of apps from each platform to generate a sample of 132 apps (62.6% of the total sample). We oversampled the Android apps that were collected to provide a balance of Android apps that would be represented in the feature space. Moreover, oversampling is a common technique used when there is an underrepresentation of 1 class [20,21]. In addition, prior work has documented health apps that were discontinued within a 12-month time frame [22]. Our final list of apps included those that remained on the market for at least 12 months (as of May 2020). Using the criteria of meeting the 12-month time frame, content written in English, and the operationalized definition of vaccination-related apps as part of the 2 lead researchers’ additional deliberation of the apps, the final number of apps included in the study was 119. To accurately classify the apps together and individually, they were categorized according to their status of iOS, Android, or both. Although we could have included apps designated as *both* in one platform, it would have been an inaccurate representation of the apps [23,24]. To evaluate and understand the nuances of vaccination apps’ descriptive, usability, information exchange capability, and privacy features, we used a mixed methods approach to frame our work. First, we developed and evaluated a codebook and conducted a content analysis of the included apps based on the codebook. This provided us with a general insight into the categories of the codebook. Second, we conducted dimensionality reduction on the features constructed from the codebook and content analysis results. Third, we wanted to identify those features that were more important for explaining variances in the data. Finally, through k-means clustering, we clustered the apps according to the feature dimension reduction results from step 3. The following sections provide additional details for each step.

### Codebook Development

To comprehensively characterize the features of the vaccination apps retrieved, we systematically developed an inclusive codebook with 4 categories (Table 1). These 4 main categories were developed during the app screening process. These broad categories have been used in similar vaccination app–related studies [6]. A total of 10 apps were randomly selected in June 2020 to evaluate the codebook, and the results were cross-validated to ensure a moderate level of agreement between the 2 coders using percentage agreement [25]. We achieved 90% agreement regarding the selected apps. Following the establishment of a stable version of the codebook, immunization apps were evaluated according to the major categories (Table 1). The 42 features across the 4 categories in Table 1 were used to represent the feature space for our computational analysis, as detailed in the following sections.

**Table 1:** Summary of the codebook features with descriptions.

Category	Features	Description
Descriptive	App name, developer; platform (iOS, Android, or both), category in the app store (medical, health and fitness, travel, or local), size in MB, ranking in its respective category if applicable, overall star rating if applicable, age rating if applicable, and cost (completely free, free to download with in-app purchase, or paid)	These descriptive characteristics gave an overview of the immunization app and such information could generally be found on the app store’s description page without the need to download or install the app [26].
Users and diseases	Target users and target diseases; for target users, we analyzed whether the app provided information on a specific user group (eg, children, parents, women, physicians, and age group); target diseases pertained to the description of a specific disease or general information concerning vaccinations and scheduling	In this category, we evaluated the targeted users and diseases of the apps. Some apps could be used by multiple, potentially overlapping groups of adult users, such as travelers and women, for which we created a specific group with binary response only. The targeted users included the following: minors, parents, travelers, women, people of all ages, and health care providers and staff. For targeted diseases, 0 was associated with no user-defined diseases, and 1 was for specific diseases such as seasonal influenza and measles-mumps-rubella [27].
Information exchange features	Account requirement for full app functionality, information presented about specific types of vaccines, educational information about vaccination and immunization in general, immunization tracking, customization of schedule, identification of nearby vaccination clinics, reminders of upcoming vaccination events, and personalized vaccination recommendations	In this category, we further explored and quantified vaccination-related core features of the apps.
Privacy and readability	Health Insurance Portability and Accountability Act–compliance feature; presence of in-app privacy statement; presence of privacy statement in the app store; presence of multilingual (at least 2 languages) privacy statement; and the average length of the privacy content (in English) using the following 7 readability measures: Simple Measure of Gobbledygook, Flesch Reading Ease score, Gunning Fog Index, Flesch-Kincaid Grade, Coleman-Liau Index, Automated Readability Index, and Linsear Write Formula [28]	Here, we considered an important element in mobile health–related research and app development, which is privacy-related features to address privacy concerns around sensitive and private vaccine health information. These features would provide information on how user-generated data would be collected, stored, shared, and transmitted on the web and offline [29,30].

### Data Analysis

#### Overview

We analyzed and evaluated the content of the apps using the aforementioned codebook through a combination of content analysis [26], descriptive statistics, and unsupervised machine learning. First, we used principal component analysis (PCA) to reduce the feature space from our original data set. Second, the apps were clustered using the k-means algorithm in R (R Foundation for Statistical Computing). The following sections will discuss in detail how PCA and k-means clustering were used in this study.

#### PCA Process

PCA is an important preprocessing step. Prior studies have used PCA to show children’s interactions with education apps [31] and reduce the context dimensions of data from smartphone apps [32]. After coding the 119 apps based on the 42 features, we conducted PCA to reduce the dimensionality space of our data. We used the *prcomp* function in R to explain the variance that was represented by the different principal components (PCs).

After identifying the proportion of variance, we determined the value of each feature contained within each PC. We used the loading values of each PC to determine this information. These values represent the correlations between the PC and the original used features. A correlation that is close to 1 or −1 indicates how important the feature is to the component. We extracted the top 5 features for each PC with the highest variance. Using these values, we reduced the number of features to represent the apps from 42 to 10. The key idea of PCA is to reduce the number of variables in the data set but preserve as much information or representation of that information in the new data set as possible [33]. Although there is no gold standard for determining the number of features to retain from this process [34], the retained features represented important components of many apps. The retained features were used to describe the data and conduct our k-means cluster analysis.

#### K-Means Cluster Analysis

Cluster analysis is used to define classes within a set of data. Clustering can be conducted using supervised and unsupervised methods. We used the unsupervised k-means clustering method to group our apps. This clustering algorithm is well documented, with successfully separating data for analysis; moreover, it has performed similar to or better than other clustering approaches [35,36]. This method uncovers latent patterns within the data and allows us to have a better understanding of which apps are associated with each other based on the selected features. To determine the number of clusters, we used the total within-cluster sum of squares (or elbow method) [37] and the silhouette method. The total within-cluster sum of squares measures how compact the clusters are. The silhouette method [38] seeks to measure the quality of the clustering. We examined how well the feature object lies within the clustering [39]. We analyzed both methods to determine the optimal number of topics to use for our k-means clustering analysis.

### Ethical Considerations

The data used in this study satisfied two research activities that did not require IRB approval, Quality Assurance and Improvement. IRB approval is not required if the study involves the practice of program evaluation, self-assessment of programs or business practices, and other quality improvement projects where methods rather than humans are the subject of the study. It also satisfies the conditions of a pilot study where the activities are intended to refine data collection procedures – time to participate, testing survey questions, etc. where any data collected are only used to plan and/or improve a future research study.

## Results

### Overview of Categories and Features

Of the 42 features, 12 (28%) were used for the descriptive app category. Of these 12 features, 9 (75%) were used for the (targeted) users and diseases category, and 8 (67%) were used for the information exchange category. Finally, 31% (13/42) of features represented the privacy and readability category. Of the 119 apps, 53 (44.5%) were iOS apps, 55 (46.2%) were for the Android operating system, and 11 (9.2%) could be found on both platforms. The Flesch-Kincaid Grade readability score (readability tests designed to indicate how difficult the content is to understand) had an average of 6.4 (SD 6.6) for both platforms combined (Table 2) [34]. Privacy statements on iOS had an average length of 850.38 (SD 1483.42) words, whereas the privacy statements of apps on Android had an average length of 790.42 (SD 1227.05) words. The user star rating was higher for the Android apps than for the iOS apps. There was a considerable difference in the sizes of apps, with iOS apps using more space (37.54 MB) than apps supported by Android (11.48 MB).

**Table 2:** Select app features characteristics (N=119).

App features		iOS (n=53)	Android (n=55)	Both (n=11)	Total
Number of ratings, mean (SD)		13.53 (62.34)	1772.8 (8136.84)	61.91 (79.07)	831.11 (5600.44)
Size in MB, mean (SD)		37.54 (41.2)	11.48 (17)	14.4 (19.47)	23.36 (32.66)
Star rating, mean (SD)		0.83 (1.59)	2.63 (2.08)	2.71 (2.07)	1.84 (2.06)
Age rating, mean (SD)		9.34 (5.4)	2.62 (4.99)	—^a^	5.37 (6.15)
Length of privacy policy (words)^b^, mean (SD)		850.38 (1483.42)	790.42(1227.05)	874.64 (1206.42)	824.91 (1329.78)
Flesch-Kincaid Grade, mean (SD)		6.13 (6.89)	6.01 (6.38)	9.63 (6.28)	6.4 (6.6)
**HIPAA^c^ compliance, n (%)**					
	Yes	7 (13)	2 (4)	0 (0)	9 (8)
	No	46 (87)	53 (96)	13 (100)	110 (92)

^a^Not available.

^b^Several apps identified contained privacy policy statements written in a different language*;however,* some apps provided an English-translated version of the policy. All apps reviewed adopted, followed, or referenced a US-based vaccination schedule (ie, Centers for Disease Control and Prevention).

^c^HIPAA: Health Insurance Portability and Accountability Act.

### PCA Results

**Figure 1:**
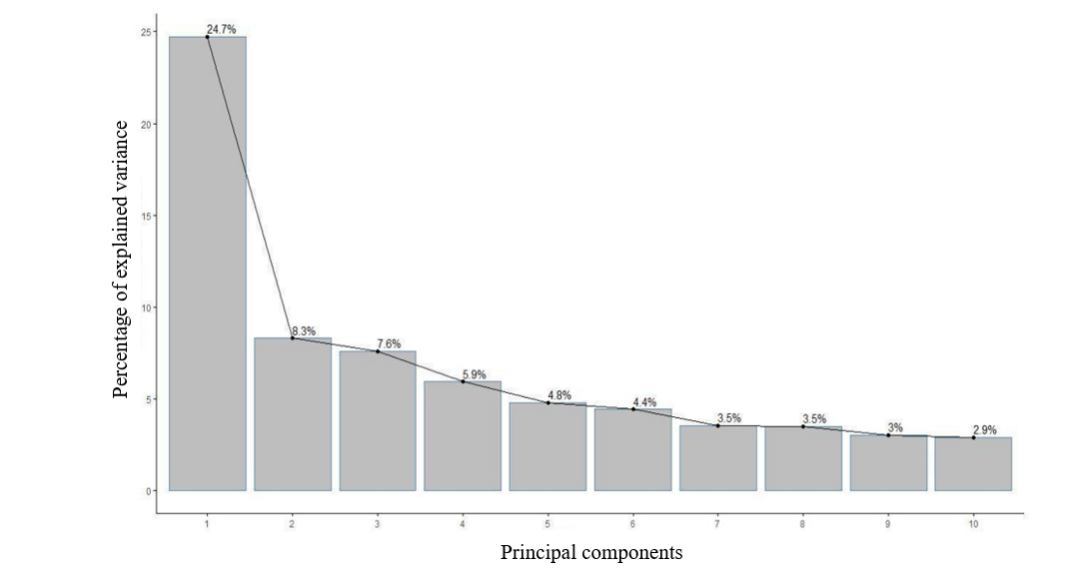
Principal component analysis showing that 24.7% of the data variance is explained by principal component 1 and 8.3% of the data variance is explained by principal component 2.

Results from the dimensionality reduction of the feature space showed that PC1 explained approximately 24.7% of the data and PC2 explained 8.3% of the data (Figure 1). The next step in our PCA involved reviewing the correlations between the PCs and the features [39]. Using the loading scores, we analyzed the values for PC1 and PC2. A review of the features for PC1 showed that the Automated Readability Index, Simple Measure of Gobbledygook, and Flesch-Kincaid Grade were the top 3 correlated features for PC1 (Textbox 1). Reminders of vaccinations, customized scheduling, and vaccination tracking were the most correlated features for PC2 based on the loading values (Textbox 1). Results from PC1 showed a high correlation between readability-related features, whereas results from PC2 showed a high correlation between customization-related features. PC2 highlights the importance of a consumer-focused approach to managing immunization schedules for children [40].

**Top 5 features correlated to their respective principal component (PC).**

**Top 5 features**

- PC1 (features related to readability)
  Automated Readability Index
  Simple Measure of Gobbledygook formula
  Flesch-Kincaid Grade
  Reading text page success
  Linsear Write Formula
- PC2 (features related to user customization)
  Reminder for vaccination
  Customized schedule
  Vaccination tracking
  Personalized recommendations
  Targeted at parents

### K-Means Cluster Analysis Results

**Figure 2:**
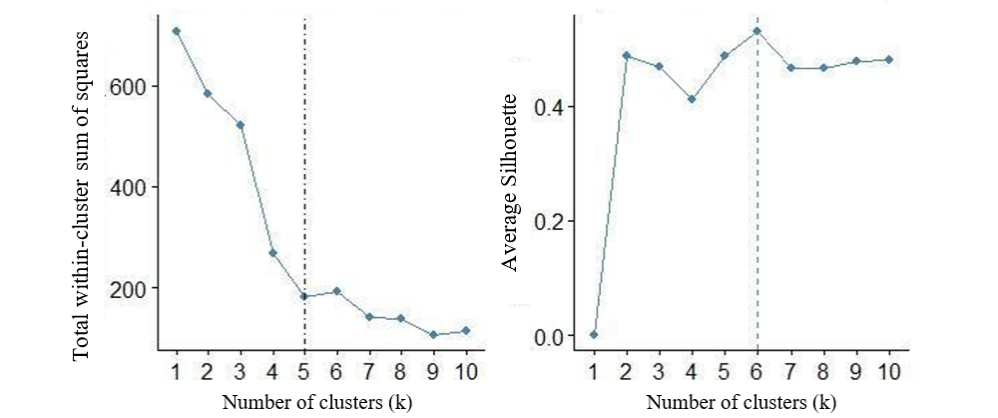
Total within-cluster sum of squares and average silhouette width. The optimal number of clusters is 5 (left) for the total within-cluster sum of squares measure and 6 (right) for the average silhouette width measure.

The top 5 features from PC1 and PC2 were used to create a cluster graph that represented the optimal number of clusters for the new feature space (Multimedia Appendix 1). In Figure 2, the dotted line represents the optimal number of clusters based on each measure. On the basis of the limited additional insight that would be derived from 6 clusters, 5 clusters were chosen as the optimal number of clusters to group the apps (Figure 2). Table 3 displays the number of apps for each cluster in accordance with selected features from the new feature space that includes the apps’ target users (*targeted parents*), *customized schedule*, and *presence of privacy policy*. Cluster 1, with 22.7% (27/119) apps, had the highest percentage of apps with a user target focused on parents. Cluster 3, with 24.4% (29/119) apps, did not offer features of customizing a schedule or the presence of a privacy policy. Cluster 1 and cluster 2, (with 59/119, 49.6% apps in total), were the only clusters with the presence of a privacy policy. Cluster 5 did not include apps found on both platforms. The specific name of each app for each cluster can be found in Multimedia Appendix 2.

**Table 3:** K-means clusters with selected new features represented (N=119).

Features		App cluster, n (%)				
		1 (n=27)	2 (n=32)	3 (n=29)	4 (n=19)	5 (n=12)
**Platform**						
	Android	12 (44)	15 (47)	13 (45)	9 (47)	6 (50)
	iOS	11 (41)	13 (41)	14 (48)	9 (47)	6 (50)
	Both	4 (15)	4 (12)	2 (7)	1 (6)	0 (0)
**Targeted parents**						
	Yes	16 (59)	4 (12)	8 (28)	6 (32)	5 (42)
	No	11 (41)	28 (88)	21 (72)	13 (68)	7 (58)
**Customized schedule**						
	Yes	25 (93)	4 (12)	0 (0)	18 (95)	10 (83)
	No	2 (7)	28 (88)	29 (100)	1 (5)	2 (17)
**Reading text page (privacy policy)**						
	Yes	27 (100)	32 (100)	0 (0)	0 (0)	0 (0)
	No	0 (0)	0 (0)	29 (100)	19 (100)	12 (100)

## Discussion

### Principal Findings

In this study, we developed a codebook to conduct a content analysis of vaccination apps and explored the use of computational approaches to identify the feature importance of vaccination apps, reduce the dimensionality of our feature space, and categorize vaccination apps using k-means clustering in an unsupervised case approach. When examining the feature importance of the 119 vaccination apps and 42 features, we found that the most important features could be categorized and explained through PC1 and PC2. For PC1, the top features found in this component were predominately associated with the *privacy and readability* category from the codebook. The category of *information exchange* had the most prominent features associated with PC2. On the basis of these results, incorporating information exchange functions and improving the readability of policy-related information should include expert involvement in vaccination app design (as denoted by clusters 1 and 2 in Table 3). Among the selected features in the cluster analysis, cluster 1 had the highest percentage of vaccination apps that provided a privacy policy, allowed a customized vaccination schedule, and targeted parents with regard to app use. Some apps that were not designed to track child vaccination information targeted parents (ie, KnowAsYouGo). Studies have detailed the lack of a government regulatory presence in the app market [6] as it relates to data privacy. Our work shows the lack of HIPAA compliance in vaccination-related apps (Table 2), although it is crucial for designers of vaccination apps in the United States to ensure agreement with HIPAA laws [41]. A transdisciplinary research approach in vaccination app design would allow for greater use by mHealth app users and opportunities to improve users’ health literacy related to vaccines. Ultimately, this would result in an overall improvement of potential information exchange with public health providers.

### App Development and Feature Analysis

mHealth technology has the potential to improve the efficiency and convenience of health care information exchange. Our findings can be categorized into two major themes: (1) features that limit the functionality of apps and (2) features that impede the overall user experience. Although most apps are moderately received by their users, based on the app rating feature, there were salient weaknesses identified through the use of PCA. This further suggests that the limitations within the reviewed vaccination apps must be addressed. On the basis of the k-means cluster analysis and the selected features, only 1 cluster of vaccination apps did not provide evidence for user vaccine schedule customization. Functionality improvements to mHealth apps could allow for a connection between patients and medical professionals to provide timely care. Systematic incorporation of information exchange features and improving policy readability would result in notable enhancements to future apps, as well as those that are currently on the market and fail to incorporate these features.

We concluded that most vaccination apps were not developed alongside health professionals. There is no standard for expert involvement in app development for any sector, and integrating medical experts in the development of mHealth apps is important, considering the increased use of mHealth technology in health care spaces [42]. Specifically related to vaccination apps, they serve as a potential tool for vaccine advocacy, administration, documentation, and monitoring success within vaccination programs. Previous research has shown a lack of engagement from public health agencies, who might have benefited from a better estimation of immunization coverage and preparedness for incoming epidemics [43]. The apps we studied were absent of any data-sharing features with public health departments, although vaccine tracking is important when monitoring vaccine programs.

### Health Literacy and Health Communication

Health literacy involves the ability of individuals to find, understand, and use services to educate themselves to make health-related decisions [44]. One of the most correlated features of the evaluated apps based on the PC1 results was the readability tests. Improved readability in mHealth apps allows for increased use among consumers and helps individuals personally educate themselves to make healthier decisions in their lives [45]. Although our readability was focused on important user privacy content, these findings also have implications for other areas of the app that require high literacy skills to operate. Our results also revealed opportunities to redesign how privacy policy information and HIPAA compliance are communicated within vaccination apps [46]. Although results from the readability measures showed that vaccination apps scored an average of 6.51 on the Flesch-Kincaid Grade readability scale, other audio and video approaches may be leveraged to improve understanding of the policy information. In reference to the information exchange theory, we see that it is crucial to have users’ personal information secured to ensure credibility. The development and redesign of the information exchange process within the apps prove to be an essential feature to adhere to policies such as HIPAA. Through these developmental improvements, we may experience an increase in vaccination app use across multiple public health sectors [47,48].

### Privacy and Security

Transferring vaccination records from paper to digital requires strict data standards and interoperability to ensure security [49]. Interoperability describes the extent to which systems can exchange and interpret shared data based on standards across health care settings. Interoperability allows for the secure exchange of medical information, which is essential for successful technological advances in health care. Less than half of the apps analyzed contained features that allowed data to be shared for personal recommendations. “Some information exchange methods involve ‘rolling out’ the electronic health records (EHRs) to unaffiliated health care organizations, creating an interface between different EHRs, or sharing a portal that allows others to view their information” [50]. Opportunities exist to develop evidence-based apps with regard to health data security and privacy concerns [51]. Credibility is a major concern of mHealth apps and may influence consumer use. This could lead to creating a systematic approach to mHealth vaccination app development and how these apps securely connect patient information with EHR systems [48].

### Strengths and Limitations

The validity of our research is upheld through a diligent acquisition and analysis of the 119 Android and iOS apps. We used 2 computational approaches to reduce the feature space and cluster our apps. Furthermore, PCA allows for the identification of specific features correlated to the larger PCs. Following the use of PCA and k-means clustering, our data provide a visual representation that is palatable for diverse audiences. The method used in our work has implications for other domain areas to examine the most important features when considering app design.

Despite a rigorous procurement and analysis of the 119 apps, our research contains several limitations. First, the apps that did not meet the 12-month time frame of representation on their respective platforms were removed from the analysis [22]. Although analyzing these apps independently was not the primary focus of this study, if included in our study, they could have affected the outcome of specific features, particularly the apps on the Android operating system. Future work should systematically evaluate apps that were discontinued during the study and compare their impact on study results. We did not observe the same issue with iOS apps. This yields potential complications for the replicability of our research in accordance with the obtained data. As a result of selecting apps exclusively from the Android and iOS app stores, there is potential for vaccination-related apps in other marketplaces to be excluded, affecting the study results. Another limitation involves bias related to the data selection process. Oversampling the Android apps creates an imbalance in the feature representation that may already be inherent to the data.

Second, this study was started in 2019, before the COVID-19 pandemic. Vaccination hesitancy along with misinformation has exacerbated vaccination uptake concerns. The landscape related to vaccination campaigns and the use of vaccination apps has changed significantly since this study started. Therefore, changes in apps that address misinformation, vaccine hesitancy, and telehealth services should be considered in future studies. Third, we used 2 exploratory machine learning approaches that can be affected by the data set size, number of features, and number of clusters. Instead of k-means clustering, the use of a hierarchical clustering method can account for grouping concerns during the cluster assignment step. Future work may incorporate other computational techniques to analyze these nuanced differences.

Finally, the researchers conducting this study are a US-based team; therefore, this research is intended to facilitate future app development. This research is also intended to supplement the further improvement of vaccination apps currently used in the United States. Not all the 119 apps featured in our research are based in the United States; this adds to the limitations of the research as it may complicate health recommendations that adhere to government and regional guidelines. Per the variation in countries where the apps are based, HIPAA compliance may not apply to other nations, and this may additionally complicate comparisons. Despite some apps being based in other countries, many internationally focused apps have followed or referenced the Centers for Disease Control and Prevention recommendations for vaccination schedules.

### Future Implications

The use of vaccines as a tool in personal and public health remains a cornerstone of disease prevention. Despite the advancement of vaccine technology and the promotion of vaccines as safe and effective, vaccine hesitancy has led to the resurgence of preventable childhood diseases. This resurgence threatens the effectiveness of vaccines as a public health tool. Technology, particularly mHealth apps, enables the intersection of public health and IT to potentially manifest positive vaccine health behaviors in individuals. Understanding the descriptive, usability, information exchange, and privacy features of these 119 mHealth apps has the potential to provide researchers and health care professionals information concerning features that should be considered when designing vaccination apps as a public health instrument.

There is conflicting literature on the overall effectiveness of mHealth apps to assist with improving vaccination coverage; however, our research yields recommendations for mHealth vaccination apps developed in the future. One recommendation is to incorporate a transdisciplinary research approach to mHealth app development, in which medical professionals, app developers, public health experts, and users can collaborate throughout the app development process. This ensures engagement from multiple stakeholders and reliable information exchange between agencies and users. As noted in the previous section, although our study was conducted before the COVID-19 pandemic, our findings could prove relevant for the ongoing monitoring of COVID-19 metrics, vaccination documentation, and beyond. One such example for mHealth apps is contact tracing for COVID-19 or serving as a liaison for information exchange between experts and users. A recent study described the most frequently installed features of contact-tracking apps as alert systems and government accountability [52]. However, the need for the exchange of information for public health purposes in contact tracing diminishes the data protection of the users. This affects users’ uptake of these mHealth apps, and prior work has shown that many apps do not include participatory user involvement with contact-tracing apps [53]. Future directions for this research include the development of a sustainable bidirectional information exchange framework for vaccination mHealth apps.

### Conclusions

We conclude that our computational method was able to identify important features of vaccination apps correlating with end user experience and categorize those apps through cluster analysis (Multimedia Appendix 1). Results from PC1 show that the top 5 features correlated with readability, and results from PC2 show that most of the top 5 features correlated with user customization. Results from our computational method provide evidence that data information exchange among different health care entities should be leveraged to provide patient-centric health care. Privacy and security concerns around the collection, storage, and sharing of health data should be addressed during the app design development process. Collaboration among multiple health stakeholders during design and development can improve the overall functionality of vaccination-related apps.

## Appendix

Multimedia Appendix 1 Completed k-means analysis using 5 clusters.

Multimedia Appendix 2 List of names for the apps that are represented in each cluster using the k-means clustering method.

## Abbreviations

EHR: electronic health record
HIPAA: Health Insurance Portability and Accountability Act
mHealth: mobile health
PC: principal component
PCA: principal component analysis

## References

[1] Awad Atheer, Trenfield Sarah J, Pollard Thomas D, Ong Jun Jie, Elbadawi Moe, McCoubrey Laura E, Goyanes Alvaro, Gaisford Simon, Basit Abdul W (2021). Connected healthcare: Improving patient care using digital health technologies. Adv Drug Deliv Rev.

[2] Mueller RC (2020). Exploring family nurse practitioners' practices in recommending mHealth apps to patients. Comput Inform Nurs.

[3] Ng YC, Alexander S, Frith KH (2018). Integration of mobile health applications in health information technology initiatives: expanding opportunities for nurse participation in population health. Comput Inform Nurs.

[4] Bhuyan SS, Lu N, Chandak A, Kim H, Wyant D, Bhatt J, Kedia S, Chang CF (2016). Use of mobile health applications for health-seeking behavior among US adults. J Med Syst.

[5] Istepanian R, Jovanov E, Zhang YT (2004). Introduction to the special section on M-Health: beyond seamless mobility and global wireless health-care connectivity. IEEE Trans Inf Technol Biomed.

[6] Facciolà A, Visalli G, Orlando A, Bertuccio MP, Spataro P, Squeri R, Picerno I, Di Pietro A (2019). Vaccine hesitancy: an overview on parents' opinions about vaccination and possible reasons of vaccine refusal. J Public Health Res.

[7] Glenn T, Monteith S (2014). Privacy in the digital world: medical and health data outside of HIPAA protections. Curr Psychiatry Rep.

[8] Bianco A, Mascaro V, Zucco R, Pavia M (2019). Parent perspectives on childhood vaccination: how to deal with vaccine hesitancy and refusal?. Vaccine.

[9] Dubé E, Laberge C, Guay M, Bramadat P, Roy R, Bettinger JA (2013). Vaccine hesitancy: an overview. Hum Vaccin Immunother.

[10] MacDonald NE, SAGE Working Group on Vaccine Hesitancy (2015). Vaccine hesitancy: definition, scope and determinants. Vaccine.

[11] Harrison EA, Wu JW (2020). Vaccine confidence in the time of COVID-19. Eur J Epidemiol.

[12] Stoyanov SR, Hides L, Kavanagh DJ, Zelenko O, Tjondronegoro D, Mani M (2015). Mobile app rating scale: a new tool for assessing the quality of health mobile apps. JMIR Mhealth Uhealth.

[13] Latella LE, McAuley RJ, Rabinowitz M (2018). Beliefs about vaccinations: comparing a sample from a medical school to that from the general population. Int J Environ Res Public Health.

[14] Simeoni R, Maccioni G, Giansanti D (2021). The vaccination process against the COVID-19: opportunities, problems and mHealth support. Healthcare (Basel).

[15] de Cock C, van Velthoven M, Milne-Ives M, Mooney M, Meinert E (2020). Use of apps to promote childhood vaccination: systematic review. JMIR Mhealth Uhealth.

[16] Balzarini F, Frascella B, Oradini-Alacreu A, Gaetti G, Lopalco PL, Edelstein M, Azzopardi-Muscat N, Signorelli C, Odone A (2020). Does the use of personal electronic health records increase vaccine uptake? A systematic review. Vaccine.

[17] Bender JL, Yue RY, To MJ, Deacken L, Jadad AR (2013). A lot of action, but not in the right direction: systematic review and content analysis of smartphone applications for the prevention, detection, and management of cancer. J Med Internet Res.

[18] Shen N, Levitan MJ, Johnson A, Bender JL, Hamilton-Page M, Jadad AA, Wiljer D (2015). Finding a depression app: a review and content analysis of the depression app marketplace. JMIR Mhealth Uhealth.

[19] Lewis TL, Boissaud-Cooke MA, Aungst TD, Eysenbach G (2014). Consensus on use of the term "App" versus "Application" for reporting of mHealth research. J Med Internet Res.

[20] Ling CX, Li C (1998). Data mining for direct marketing: problems and solutions. Proceedings of the Third International Conference on Knowledge Discovery and Data Mining.

[21] He H, Garcia EA (2009). Learning from imbalanced data. IEEE Trans Knowl Data Eng.

[22] Tabi K, Randhawa AS, Choi F, Mithani Z, Albers F, Schnieder M, Nikoo M, Vigo D, Jang K, Demlova R, Krausz M (2019). Mobile apps for medication management: review and analysis. JMIR Mhealth Uhealth.

[23] Wang RJ (2020). Branded mobile application adoption and customer engagement behavior. Comput Human Behav.

[24] Ebone A, Tan Y, Jia X (2018). A performance evaluation of cross-platform mobile application development approaches. Proceedings of the IEEE/ACM 5th International Conference on Mobile Software Engineering and Systems.

[25] McHugh ML (2012). Interrater reliability: the kappa statistic. Biochem Med (Zagreb).

[26] Jimenez G, Lum E, Car J (2019). Examining diabetes management apps recommended from a Google search: content analysis. JMIR Mhealth Uhealth.

[27] Yin H, Wardenaar KJ, Wang Y, Wang N, Chen W, Zhang Y, Xu G, Schoevers RA (2020). Mobile mental health apps in China: systematic app store search. J Med Internet Res.

[28] Robillard JM, Feng TL, Sporn AB, Lai JA, Lo C, Ta M, Nadler R (2019). Availability, readability, and content of privacy policies and terms of agreements of mental health apps. Internet Interv.

[29] Arora S, Yttri J, Nilse W (2014). Privacy and security in mobile health (mHealth) research. Alcohol Res.

[30] Aljedaani B, Babar MA (2021). Challenges with developing secure mobile health applications: systematic review. JMIR Mhealth Uhealth.

[31] Crescenzi-Lanna L (2020). Emotions, private speech, involvement and other aspects of young children's interactions with educational apps. Comput Human Behav.

[32] Sarker IH, Abushark YB, Khan AI (2020). ContextPCA: predicting context-aware smartphone apps usage based on machine learning techniques. Symmetry.

[33] Jolliffe IT, Cadima J (2016). Principal component analysis: a review and recent developments. Philos Trans A Math Phys Eng Sci.

[34] Steinley D (2006). K-means clustering: a half-century synthesis. Br J Math Stat Psychol.

[35] Kruse J, Toledo P, Belton TB, Testani EJ, Evans CT, Grobman WA, Miller ES, Lange EM (2021). Readability, content, and quality of COVID-19 patient education materials from academic medical centers in the United States. Am J Infect Control.

[36] Dobbins C, Rawassizadeh R (2015). Clustering of physical activities for quantified self and mHealth applications. Proceedings of the 2015 IEEE International Conference on Computer and Information Technology; Ubiquitous Computing and Communications; Dependable, Autonomic and Secure Computing; Pervasive Intelligence and Computing.

[37] Syakur MA, Khotimah BK, Rochman EM, Satoto BD (2018). Integration K-means clustering method and elbow method for identification of the best customer profile cluster. IOP Conference Series: Materials Science and Engineering, Volume 336, The 2nd International Conference on Vocational Education and Electrical Engineering.

[38] Rousseeuw PJ (1987). Silhouettes: a graphical aid to the interpretation and validation of cluster analysis. J Comput Appl Math.

[39] Burger SV (2018). Introduction to Machine Learning with R.

[40] Seeber L, Conrad T, Hoppe C, Obermeier P, Chen X, Karsch K, Muehlhans S, Tief F, Boettcher S, Diedrich S, Schweiger B, Rath B (2017). Educating parents about the vaccination status of their children: a user-centered mobile application. Prev Med Rep.

[41] Al Ayubi SU, Pelletier A, Sunthara G, Gujral N, Mittal V, Bourgeois FC (2016). A mobile app development guideline for hospital settings: maximizing the use of and minimizing the security risks of "bring your own devices" policies. JMIR Mhealth Uhealth.

[42] Pagoto S, Schneider K, Jojic M, DeBiasse M, Mann D (2013). Evidence-based strategies in weight-loss mobile apps. Am J Prev Med.

[43] Fadda M, Galimberti E, Fiordelli M, Romanò L, Zanetti A, Schulz PJ (2017). Effectiveness of a smartphone app to increase parents' knowledge and empowerment in the MMR vaccination decision: a randomized controlled trial. Hum Vaccin Immunother.

[44] Liu C, Wang D, Liu C, Jiang J, Wang X, Chen H, Ju X, Zhang X (2020). What is the meaning of health literacy? A systematic review and qualitative synthesis. Fam Med Community Health.

[45] Dunn Lopez K, Chae S, Michele G, Fraczkowski D, Habibi P, Chattopadhyay D, Donevant SB (2021). Improved readability and functions needed for mHealth apps targeting patients with heart failure: an app store review. Res Nurs Health.

[46] Powell AC, Singh P, Torous J (2018). The complexity of mental health app privacy policies: a potential barrier to privacy. JMIR Mhealth Uhealth.

[47] Willcox JC, Dobson R, Whittaker R (2019). Old-fashioned technology in the era of "bling": is there a future for text messaging in health care?. J Med Internet Res.

[48] Al-Azzam MK, Bader Alazzam M, Khalid al-Manasra M (2019). MHealth for decision making support: a case study of eHealth in the public sector. Int J Adv Comput Sci Appl.

[49] Maurer W, Seeber L, Rundblad G, Kochhar S, Trusko B, Kisler B, Kush R, Rath B, Vienna Vaccine Safety Initiative (2014). Standardization and simplification of vaccination records. Expert Rev Vaccines.

[50] Everson J (2017). The implications and impact of 3 approaches to health information exchange: community, enterprise, and vendor-mediated health information exchange. Learn Health Syst.

[51] Mosa AS, Yoo I, Sheets L (2012). A systematic review of healthcare applications for smartphones. BMC Med Inform Decis Mak.

[52] Kolasa K, Mazzi F, Leszczuk-Czubkowska E, Zrubka Z, Péntek M (2021). State of the art in adoption of contact tracing apps and recommendations regarding privacy protection and public health: systematic review. JMIR Mhealth Uhealth.

[53] Osmanlliu E, Rafie E, Bédard S, Paquette J, Gore G, Pomey MP (2021). Considerations for the design and implementation of COVID-19 contact tracing apps: scoping review. JMIR Mhealth Uhealth.

