# Supplementary figures and images for: Separating Features From Functionality in Vaccination Apps: Computational Analysis
Source: JMIR Form Res. 2022 Oct 11;6(10):e36818. doi: 10.2196/36818 (PMC9597419; doi:10.2196/36818)

**
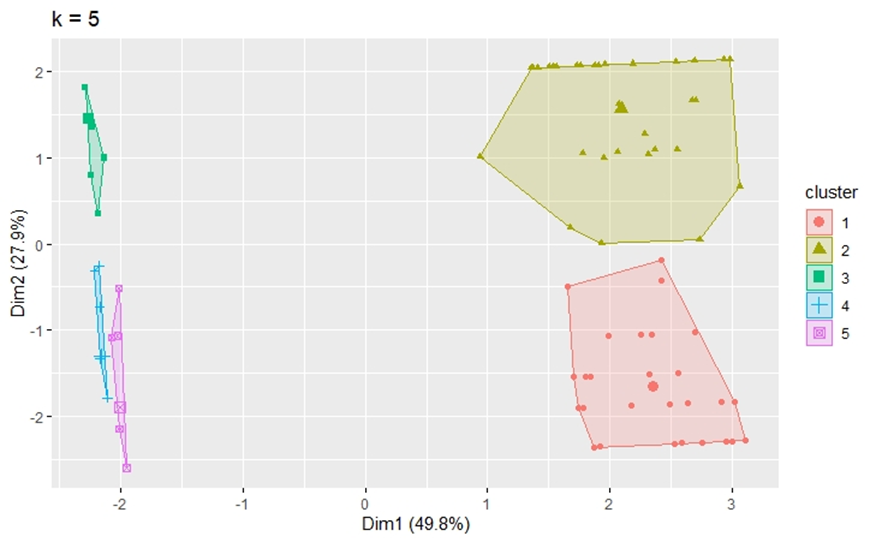
**

Supplement: Multimedia Appendix 1 [file formative_v6i10e36818_app1.docx]
