# Supplementary material for: Separating Features From Functionality in Vaccination Apps: Computational Analysis
Source: JMIR Form Res. 2022 Oct 11;6(10):e36818. doi: 10.2196/36818 (PMC9597419; doi:10.2196/36818)
Supplement: Multimedia Appendix 2 [file formative_v6i10e36818_app2.docx]

|  | Platform | Cluster | Target at Parents | Customized Schedule | Reading Text Page Success (Privacy Policy) |
| --- | --- | --- | --- | --- | --- |
| CANImmunize | Android | 1 | 0 | 1 | 1 |
| CDC TravWell | Android | 1 | 0 | 1 | 1 |
| ChartNotes | iOS | 1 | 0 | 1 | 1 |
| Child Vaccination Schedule | Android | 1 | 1 | 1 | 1 |
| Healthy Children | iOS | 1 | 1 | 1 | 1 |
| Immunization Planner | Android | 1 | 1 | 1 | 1 |
| Immunize | Android | 1 | 1 | 1 | 1 |
| Immunize India | Android | 1 | 1 | 1 | 1 |
| iVaccination India | Both | 1 | 0 | 1 | 1 |
| KnowAsYouGo | iOS | 1 | 1 | 0 | 1 |
| My Baby's List | iOS | 1 | 1 | 1 | 1 |
| mychart | Android | 1 | 1 | 1 | 1 |
| Pediatric Oncall | iOS | 1 | 0 | 1 | 1 |
| ReadyVax | iOS | 1 | 0 | 1 | 1 |
| Redbook | Both | 1 | 1 | 1 | 1 |
| SJBaby | iOS | 1 | 0 | 1 | 1 |
| Thrive | Both | 1 | 1 | 1 | 1 |
| Vac On Time | iOS | 1 | 0 | 1 | 1 |
| VacciDate | iOS | 1 | 1 | 1 | 1 |
| Vaccine Awareness Vac Aware | Android | 1 | 1 | 0 | 1 |
| Vaccine Reminder | Both | 1 | 1 | 1 | 1 |
| VaccineApp | Android | 1 | 1 | 1 | 1 |
| Vaccines on the Go | iOS | 1 | 0 | 1 | 1 |
| Vaxini vaccines vacci0tion | Android | 1 | 1 | 1 | 1 |
| VDD Baby | Android | 1 | 1 | 1 | 1 |
| vImmune | Android | 1 | 0 | 1 | 1 |
| ZibdyHealth | iOS | 1 | 0 | 1 | 1 |
| AAP Red Book | Android | 2 | 0 | 1 | 1 |
| AccuVax | iOS | 2 | 0 | 0 | 1 |
| ACOG | Both | 2 | 0 | 0 | 1 |
| ACOG App | Android | 2 | 0 | 0 | 1 |
| CDC Vaccine Schedules | Android | 2 | 0 | 1 | 1 |
| CDC Yellow Book 2018 | Android | 2 | 0 | 0 | 1 |
| Contemporary Pediatrics | iOS | 2 | 0 | 0 | 1 |
| Clear – Health, travel, sports | Android | 2 | 0 | 0 | 1 |
| Help+ | iOS | 2 | 0 | 0 | 1 |
| HPV Vaccine: Same way same day | iOS | 2 | 1 | 0 | 1 |
| Immunization Schedule | Android | 2 | 1 | 0 | 1 |
| Infant Enlighten Training | iOS | 2 | 0 | 0 | 1 |
| Johns Hopkins Antibiotic Guide | iOS | 2 | 0 | 0 | 1 |
| Kids Wellness Tracker | iOS | 2 | 1 | 0 | 1 |
| MEDIvate | iOS | 2 | 0 | 1 | 1 |
| Medscape | Android | 2 | 0 | 0 | 1 |
| MedWatcher | Android | 2 | 0 | 0 | 1 |
| Microsoft Health Vault | iOS | 2 | 0 | 0 | 1 |
| MMWR Express | Both | 2 | 0 | 0 | 1 |
| MyVaccines | iOS | 2 | 0 | 0 | 1 |
| Pediatrics Genius | iOS | 2 | 0 | 0 | 1 |
| Pneumococcal Vaccines | Both | 2 | 0 | 0 | 1 |
| PneumoRecs Vax Advisor | Both | 2 | 0 | 0 | 1 |
| RxNeed | iOS | 2 | 0 | 0 | 1 |
| Shots Immunizations | Android | 2 | 0 | 0 | 1 |
| Sprout Baby | Android | 2 | 0 | 0 | 1 |
| The Vaccine Handbook App | iOS | 2 | 0 | 0 | 1 |
| Travel Smart (Canada) | Android | 2 | 0 | 0 | 1 |
| Vaccination chart for children | Android | 2 | 0 | 0 | 1 |
| Vaccine & Vaccination Schedules | Android | 2 | 1 | 0 | 1 |
| Vaccine Schedule | Android | 2 | 0 | 0 | 1 |
| Women's Health Immunization | Android | 2 | 0 | 1 | 1 |
| Baby Vaccination | Android | 3 | 0 | 0 | 0 |
| BabyVaccine | Android | 3 | 0 | 0 | 0 |
| Calendario de Vacunacion ARG | Both | 3 | 1 | 0 | 0 |
| Child Vaccine | Android | 3 | 1 | 0 | 0 |
| E-HPV | iOS | 3 | 0 | 0 | 0 |
| Flu Clinics | iOS | 3 | 0 | 0 | 0 |
| Flu Vaccine Finder | Android | 3 | 0 | 0 | 0 |
| Vaccine Reactions | Android | 3 | 0 | 0 | 0 |
| Growing Trees | iOS | 3 | 1 | 0 | 0 |
| Healthy Kids AZ | iOS | 3 | 0 | 0 | 0 |
| HPV Cancer Free | iOS | 3 | 1 | 0 | 0 |
| HPV Vaccine | Both | 3 | 0 | 0 | 0 |
| Immunization Summary | iOS | 3 | 0 | 0 | 0 |
| Immunization Tracker | iOS | 3 | 0 | 0 | 0 |
| ImmunizationLog | iOS | 3 | 1 | 0 | 0 |
| Ivaccine | Android | 3 | 0 | 0 | 0 |
| MothertoBaby | iOS | 3 | 1 | 0 | 0 |
| NormalChild: Health Record | iOS | 3 | 1 | 0 | 0 |
| The Travel Clinic | iOS | 3 | 0 | 0 | 0 |
| Vaccine | Android | 3 | 0 | 0 | 0 |
| Vaccine Adverse Reactions | iOS | 3 | 0 | 0 | 0 |
| Vaccine Consent Forms App | Android | 3 | 0 | 0 | 0 |
| Vaccine Guidance | Android | 3 | 0 | 0 | 0 |
| Vaccines Guide | Android | 3 | 0 | 0 | 0 |
| Vaccine Knowledge | iOS | 3 | 0 | 0 | 0 |
| Vaccine Scheduler | Android | 3 | 1 | 0 | 0 |
| Vaccines Information | Android | 3 | 0 | 0 | 0 |
| Vaccine Monitor | Android | 3 | 0 | 0 | 0 |
| vicvax | iOS | 3 | 0 | 0 | 0 |
| Baby 2.0 | iOS | 4 | 1 | 1 | 0 |
| Biopure | iOS | 4 | 0 | 1 | 0 |
| Cartao Vacicao Blockchain (now called Immunization Protocol Blockchain) | Both | 4 | 0 | 1 | 0 |
| Child Health Docs | iOS | 4 | 0 | 1 | 0 |
| Child Heath Log | iOS | 4 | 0 | 1 | 0 |
| Child Medical History | iOS | 4 | 1 | 1 | 0 |
| Family Medical History | iOS | 4 | 0 | 1 | 0 |
| Kid Vaccines | iOS | 4 | 1 | 1 | 0 |
| MatImms | Android | 4 | 1 | 1 | 0 |
| My Immunizations | iOS | 4 | 0 | 1 | 0 |
| MyVaxIndia | Android | 4 | 0 | 1 | 0 |
| Vaccination Book | Android | 4 | 0 | 1 | 0 |
| Vaccination Records | Android | 4 | 0 | 1 | 0 |
| Vaccine Global | Android | 4 | 0 | 1 | 0 |
| Vaccine Record for Travellers | iOS | 4 | 0 | 1 | 0 |
| Vaccine Time | Android | 4 | 0 | 1 | 0 |
| Vaccines | Android | 4 | 0 | 1 | 0 |
| Vaccinise | Android | 4 | 1 | 1 | 0 |
| VaccTrack | Android | 4 | 1 | 0 | 0 |
| Baby Vaccine Scheduler | Android | 5 | 0 | 1 | 0 |
| BabyAgenda | iOS | 5 | 0 | 1 | 0 |
| Babymate | iOS | 5 | 0 | 1 | 0 |
| Doting Mom | iOS | 5 | 1 | 1 | 0 |
| Go4Vaccine | Android | 5 | 1 | 1 | 0 |
| My Travel Health | iOS | 5 | 0 | 1 | 0 |
| Vaccination Reminder | Android | 5 | 1 | 1 | 0 |
| Vaccination Scheduler | Android | 5 | 1 | 1 | 0 |
| Vaccinations Reminder | Android | 5 | 0 | 1 | 0 |
| Children Vaccination Chart | Android | 5 | 0 | 0 | 0 |
| Women's Health Diary | iOS | 5 | 1 | 1 | 0 |
| Your Travel Health | iOS | 5 | 0 | 0 | 0 |
